# Experimental and Numerical Study of the Plasma Arc Melting of Titanium Alloys: Application to the Removal of High Density Inclusions (HDIs)

**DOI:** 10.3390/ma18092051

**Published:** 2025-04-30

**Authors:** Jean-Pierre Bellot, Widad Ayadh, Jean-Sébastien Kroll-Rabotin, Raphaël Marin, Jérôme Delfosse, Amandine Cardon, Alessia Biagi, Stéphane Hans

**Affiliations:** 1 Institut Jean Lamour—UMR CNRS 7198, LabEx DAMAS, Université de Lorraine, 54000 Nancy, France; widad.ayadh@irt-m2p.fr (W.A.); jean-sebastien.kroll-rabotin@univ-lorraine.fr (J.-S.K.-R.); 2 IRT M2P, 4 rue Augustin Fresnel, 57070 Metz, France; raphael.marin@irt-m2p.fr; 3 Safran Tech, Rue des Jeunes Bois, 78772 Châteaufort, France; jerome.delfosse@safrangroup.com (J.D.); amandine.cardon@safrangroup.com (A.C.); 4 Aubert & Duval, Site Les Ancizes, BP1, 63770 Les Ancizes CEDEX, France; alessia.biagi@aubertduval.com (A.B.); stephane.hans@aubertduval.com (S.H.)

**Keywords:** titanium alloys, recycling, plasma melting, inclusion, simulation

## Abstract

Titanium alloys are increasingly used in aeronautical applications, a sector that requires highly controlled materials. In particular, inclusion cleanliness is a necessary and mandatory condition for safe use in aeronautical components. During the production and processing of titanium alloys, inclusions are likely to appear, in particular high-density inclusions (HDIs) originate from refractory metals such as molybdenum or tungsten carbide. Plasma Arc Melting–Cold Hearth Remelting (PAMCHR) is one of the most effective recycling and refining process for titanium alloys. Firstly, this work reports the thermal modeling of the melting of raw materials in the melting crucible and a complete 3D numerical simulation of the thermo-hydrodynamic behavior of the metal flow in the PAMCHR furnace, based on the software Ansys-Fluent CFD V21.1. Simulation results are presented for a 100 kg/h melting test performed in a pilot furnace with a comparison between the measured and calculated pool profiles and residence time distributions that show satisfactory agreements. Additionally, a Lagrangian calculation of particle trajectories in the liquid metal pool is also performed and insemination of HDIs in the pilot furnace has been tested. Both numerical and experimental tests demonstrate the inclusion removal in the melting crucible.

## 1. Introduction

Titanium is a metal widely used in the aerospace industry due to its low density compared to steels and superalloys, as well as its excellent mechanical properties, including toughness, corrosion resistance, and ductility. In addition to its classification by the EU as a critical material, the high production cost of titanium—primarily due to the complex and energy-intensive process of manufacturing titanium sponge—makes recycling particularly economically viable [1,2].

Currently, titanium recycling mainly focuses on alloy scraps generated during the manufacturing of aircraft and engine components, such as swarf, cutting chips, and turnings [3]. Beyond its economic benefits, recycling also enhances environmental sustainability by reducing waste, electrical energy consumption, and associated CO_2_ emissions.

To either compete with or complement Vacuum Arc Remelting (VAR), an alternative recycling technology utilizing water-cooled, flat-bottomed copper crucibles emerged in the 1990s, offering lower production costs compared to VAR, higher recycling possibility, and better inclusion removal potential [4,5]. Depending on the heat source, this technology is categorized into two processes: Electron Beam Cold Hearth Refining (EBCHR) and Plasma Arc Melting–Cold Hearth Refining (PAMCHR). The EBCHR process employs electron guns in a vacuum environment to ensure proper beam operation, while PAMCHR uses plasma torches under an inert gas atmosphere (Argon or Helium).

**Figure 1:**
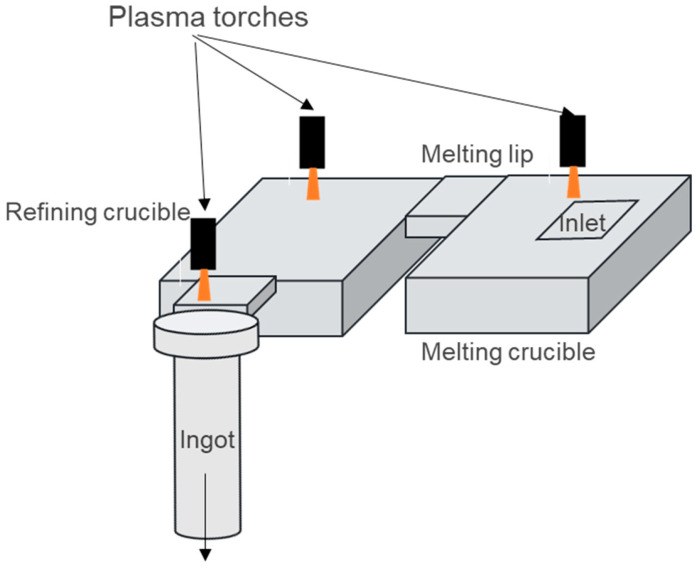
Schematic of remelting processes with water-cooled crucibles and PAMCHR.

For these processes to reach the alloy composition, titanium scraps are melted with some virgin titanium sponge and master alloys in the first melting chamber of the crucible, as illustrated in Figure 1. The liquid metal then flows through a refining crucible before finally reaching a mold casting crucible, where it solidifies into a secondary ingot.

Characteristics of the EB process include a relatively quiet molten pool and a quasi-steady distribution of the input heat. But the vacuum environment leads to evaporation losses of the alloy components with a high vapor pressure, such as Al and Cr [6,7,8]. On the contrary, the very low evaporation is the main advantage of the PAMCHR process against EBCHR, which uses plasma torches as the heat source (operating pressure range between 0.4 and 1 bar).

Despite industrial interest in the PAMCHR process over the past twenty years, the mathematical modeling required to optimize the process remains underdeveloped. A more detailed review of the literature is available at [9]. Modeling began in the late 1990s with the work of Huang et al. [10], including the blowing effect of the plasma jet on the bath surface. Subsequently, in 2013, Xu et al. [11] developed a CFD model based on Ansys-Fluent software V21.1 applied to Ti-Al intermetallic remelting. More recently, in 2018, the Department of Materials Engineering at UBC [12,13] focused their experimental and modeling work on energy transfer from arc plasma to liquid bath.

Inclusion removal is a critical concern for titanium alloys used in aerospace applications. Two types of inclusions can be distinguished based on their density—and consequently—their chemical composition: low-density inclusions (LDIs) and high-density inclusions (HDIs) [14]. Both are exogenous, meaning they form before the remelting process, and their occurrence is extremely rare—some studies estimate only one inclusion per 5000 tons [15]. Due to their diverse origins [16], these particles can vary in size from a few hundred micrometers to several millimeters. If not eliminated during remelting, these inclusions can cause fractures during extended fatigue cycles, such as those experienced by rotating jet engine components. For obvious safety reasons, an alloy free of inclusions is compulsory for engine manufacturing, and remelting processes are specifically designed to remove these two main types of exogenous inclusions. In this paper, we focus exclusively on HDIs, while LDIs will be addressed in a future publication.

HDIs include refractory metals such as tungsten (W), molybdenum (Mo), and tantalum (Ta), along with carbides like tungsten carbide (WC) [17,18]. These defects primarily originate from machining tool fragments. Consequently, recycling machining off-cuts are a major source of HDIs in the recycling materials. Due to their high melting points and slow dissolution rates, HDIs pose a significant challenge. They do not melt and, as noted by Bomberger and Froes [19], typically sink to the bottom of crucibles during remelting, where they dissolve slowly. Yamanaka and Ichihashi [20] investigated the sinking and dissolution behavior of Ta and Mo cylinders in a titanium pool during VAR, using a 100 mm diameter mold. They reported dissolution rates of 0.5 μm/s for Ta and 0.7 μm/s for Mo and show that HDIs might survive the VAR process. In 2010, Ghazal et al. [21] examined the behavior of HDIs (W and Mo) in titanium and titanium alloy (Ti64 and Ti17) baths melted by an electron beam, measuring dissolution rates ranging from 1.6 to 8 µm/s. Finally, Xu et al. [22] employed CFD simulations to demonstrate that HDIs can be removed by trapping them at the bottom of PAM crucibles due to their relative density. The residence time in the liquid bath before entrapment is short, always under four seconds.

**Figure 2:**
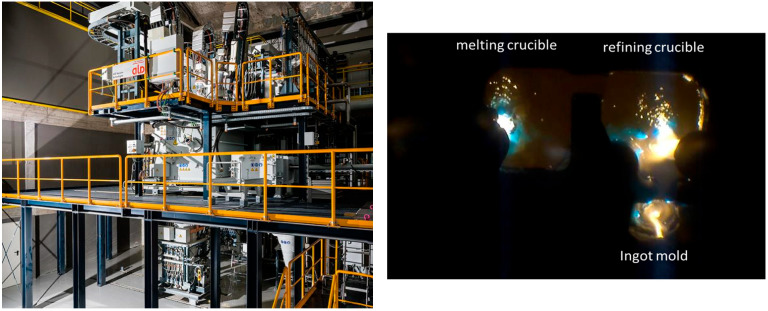
IRT-M2P’ pilot furnace (**left**) and top view of the crucibles during processing (**right**).

The TIARE (“TItanium Aerospace REcycling”) project brings together the authors of this paper and aims to support the titanium recycling industry by enhancing the understanding of the recycling process. To this end, IRT-M2P operates a high-capacity pilot furnace equipped with three torches, delivering a total power of 1.2 MW and enabling melting rates of up to 150 kg/h of Ti64. The melting and refining crucibles, along with the ingot mold, are arranged in a U-shape, as in the schematics shown in Figure 1 and Figure 2. As part of the TIARE project, we conducted tests to measure liquid bath profiles, residence time distribution, and seeding with synthetic HDIs. Additionally, a comprehensive PAM3D phenomenological model of the PAMCHR furnace was developed, building on an initial version presented in [9], which modeled heat and momentum transfers between the plasma jet and the liquid surface. PAM3D is currently being further developed at Institut Jean Lamour using the commercial CFD software Ansys-Fluent V21.1. In this new version of the numerical code, both the melting and refining crucibles are modeled, with particular focus on the raw material melting stage. Finally, the dynamic behavior of HDIs is simulated using a Lagrangian approach. The results of the PAM3D model are compared with new experimental data from pilot furnace tests, thus making this paper particularly original.

## 2. Methods

### 2.1. Trials Using the Pilot Furnace

**Figure 3:**
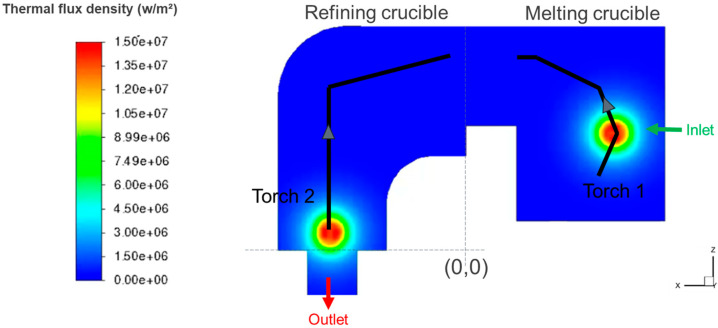
Patterns of the two torches (black lines) and instantaneous heat flux density for a given torch position.

Three series of tests were carried out using the IRT-M2P pilot furnace, with identical operating conditions, i.e., an electrical power of 358 kW per torch (U = 203 V and I = 1765 A). The scanning pattern of each torch is shown in Figure 3, each torch having a period of 15 s. Torch 1 spends most of its time on the melting zone with the aim of melting the solid input charge.

#### 2.1.1. Shape of the Liquid Pool

An initial comparison between the simulation and an experimental test is based on estimating the liquid bath height in the melting and refining crucibles. To facilitate macrographic analysis of the liquid front, the steady-state melting of Ti64 is replaced with TiCp (Commercially Pure), creating a chemical distinction between the liquid bath and the skull. The skull was sectioned at six different locations and in two directions to compare the liquid pool profiles.

#### 2.1.2. RTD Measurement

To experimentally measure the Residence Time Distribution (RTD), a copper marker was added to the titanium load. First, a 37 kg Ti64 bar was melted to establish a quasi-stationary regime. Then, a second Ti64 bar, containing a 127 g copper cube inserted into a drilled hole, was introduced. After melting, the solidified ingot (1233 mm in height and 150 mm in diameter) was cut in half and chemically analyzed through sampling along two longitudinal profiles: one at the center (along the ingot’s axis) and the other at the periphery. The copper mass fraction was determined using Inductively Coupled Plasma (ICP) spectrometry.

#### 2.1.3. HDI Insemination

**Figure 4:**
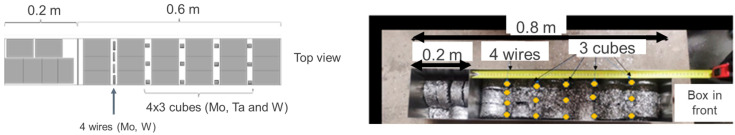
Arrangement of the HDIs in the Ti64 briquette box (**left**), and photograph of the box after deposition of the first briquette layer (**right**).

A test was carried out with compacted sponges (called here briquette) seeded with HDIs. In the compact box, 5 mm cubes of Mo, W, and Ta were placed (appearing in yellow—one in the center and one on each side of the box) at four different distances from the front of the box, as seen in the top view of Figure 4. At the outer edge of the box (from 0.60 m or three-quarters of the box), unseeded briquettes were placed. The box was then filled with a second layer of briquettes over the first layer. The total weight of the filled box was 39 kg.

### 2.2. Mathematical Modeling

To account for the effects of torch sweeping over the bath, PAM3D models the metal liquid flow in the crucibles in transient and turbulent regimes. To simplify and reduce complexity, we apply the following assumptions:

(i) The surface of the liquid bath is considered to be flat; deformation of the surface under the plasma torch jet is neglected. The torch blast and its action on the bath are taken into account through a shear stress τ_s_ exerted on the bath surface and distributed as a function of the distance to the point of impact of each torch. The value of this stress has been estimated from a simplified 2D model of the plasma jet [9]. This 2D model and the work provided by Huang et al. [10] demonstrate that the deformation of the free surface can be neglected in a first stage of modeling.
(ii) The effects of the electromagnetic forces are neglected. The electric current transferred between the plasma torch and the liquid bath induces an electromagnetic force. Using a 2D model, a comparison between electromagnetic and natural convection forces showed the predominance of the latter, and consequently the neglect of electromagnetic forces [23].
(iii) The flow of liquid metal is mainly controlled by the movement of the torches. The torches move cyclically along a continuous trajectory with a configurable period. The heat flux and parietal stress distributions due to the torch jets follow a radial distribution around the moving impact point, and their expressions are detailed in [9].

#### 2.2.1. Governing Transport Equations

To avoid making this paper excessively long, the set of governing transport equations (mass, momentum and heat) are not reported here but are extensively described in references [9,23,24]. The reader can find a detailed description of the governing equations in these references. The momentum and heat equations are coupled in particular, but not exclusively, by thermal natural convection modeled through the Boussinesq assumption:

(1) $$\rho =\rho_{0}-\beta_{T}\rho_{0}(T-T_{0})$$

Because the crucibles are water-cooled, a solid phase called “skull” develops on contact with the cold walls, as shown later. The Kozeny–Carman relation is applied to calculate the permeability *K* in the mushy zone [25]. The volume fraction of liquid called *g_l_* is expressed as a linear function of the temperature between the solidus (T_sol_) and liquidus (T_liq_) of the alloy. Accordingly, the permeability *K* falls to zero when the temperature reaches the solidus, leading to a zero velocity in the solid phase.

(2) $$K=K_{0}\frac{g_{l}^{3}}{{\left(1-g_{l}\right)}^{2}}$$

The turbulence in the liquid pool was described with the realizable k-ε model [24], where the production of the turbulent kinetic energy takes into account velocity gradient and buoyancy. This model has become the industry standard for many kinds of engineering problems, especially within the metallurgy industry.

#### 2.2.2. Boundary Conditions

**Figure 5:**
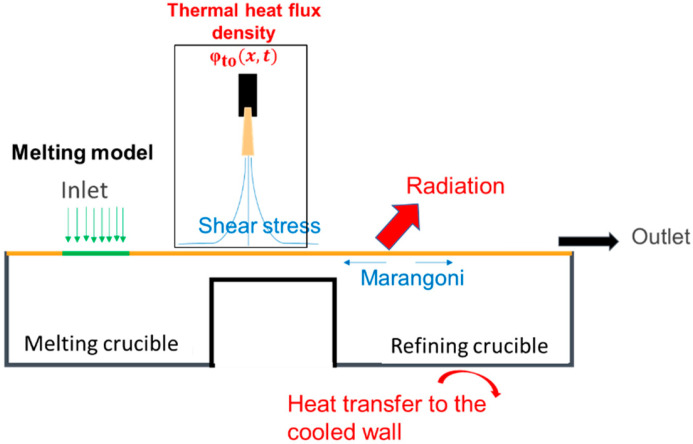
Boundary conditions applied.

The boundary conditions are summarized in Figure 5, where particular attention has been given to transfers between the plasma and the metal surface [9].

The upper surface radiates toward the water-cooled furnace walls. The radiation is expressed by the Stefan–Boltzmann relation using the emissivity ε_m_ corresponding to the state (solid or liquid) of the metal on the surface:

(3) $$\varphi_{\mathrm{ray}}^{\mathrm{up}}(T)=\sigma_{\mathrm{sb}}\varepsilon_{m}\left(T^{4}-T_{\mathrm{fur}}^{4}\right)$$

with σ_sb_ the Stefan–Boltzmann constant, T the surface temperature, and ε_m_ = 0.23 for liquid surfaces and 0.60 for solid [26]. T_fur_ is the temperature of the water-cooled furnace walls, assumed to be uniform and equal to 100 °C.

The heat flux density contributed by the torches to the bath surface φ_to_ is calculated from the sum of the electrical α_elec_φ_elec_, convective α_conv_φ_conv_, and radiative α_ray_φ_ray_ contributions of each torch, defined by the following equation:

(4) $$\varphi_{\mathrm{to}}(x,t)=\sum_{i}\left[\alpha_{conv}\varphi_{conv}\left(\left|\vec{x}-{\vec{x}}_{i}(t)\right|\right)+\alpha_{elec}\varphi_{elec}\left(\left|\vec{x}-{\vec{x}}_{i}(t)\right|\right)+\alpha_{\mathrm{ray}}\varphi_{\mathrm{ray}}\left(\left|\vec{x}-{\vec{x}}_{i}(t)\right|\right)\right]\eta_{to}U_{i}(t)I_{i}(t)$$

U_i_ and I_i_ are, respectively, the voltage and current at which each torch i is operated, which vary with time, as does the position x_i_(t) of each torch’s impact point. η_to_ is the torch efficiency, i.e., the ratio between heat power and electrical power. It is estimated to be 0.66 [23]. The shape of the φ_conv_, φ_elec_, and φ_ray_ distributions are detailed in [9] and their relative contributions (α coefficients) are noted in Table 1.

A Fourier condition is applied to model the heat resistance between the solidified alloy and copper wall:

(5) $$\varphi_{\mathrm{wall}}=h_{\mathrm{wall}}\left(T_{p}-T_{\mathrm{wall}}\right)$$

where T_wall_ is assumed equal to 100 °C. The value h_wall_ = 500 W/m^2^/K is then used as the averaged value, which was reported in [27] for the simulation of PAMCHR.

On the liquid surface, a shear stress $\tau_{s}$ is calculated in the region of the arc plasma blast. It varies radially around the impact center of each torch, as follows:

(6) $$\tau_{s}(r)=f(r)\tau_{\max}$$

The function f(r) is zero at the stopping point of the plasma flow, giving a value of zero for the stress. The value of τ_max_ = 10 Pa is retained in the model. The distribution function and the numerical values of the coefficients are given and justified in [9].

Since the surface tension of liquid metal varies with temperature, temperature gradients create surface tension differences, leading to tangential stress—known as the “Marangoni effect”. This phenomenon can be described using the following equations:

(7) $$\tau_{yx}=\frac{\partial \sigma }{\partial T}{\left(\frac{\partial T}{\partial x}\right)}_{s}\;and\;\tau_{zx}=\frac{\partial \sigma }{\partial T}{\left(\frac{\partial T}{\partial z}\right)}_{s}$$

In the model, the variation of surface tension with temperature (∂σ/∂T) is assumed constant and is provided as input to the simulations.

#### 2.2.3. Melting Model

The melting model is applied to each cell node P of the pre-defined inlet zone (S_inlet_). The melting rate ${\dot{m}}_{p}$ is calculated for each cell P under this feed surface, based on an enthalpy balance, as follows:

(8) $$\left(\varphi_{to}(x_{p},t)-\varphi_{ray}^{up}(x_{p},t)\right)S_{n}={\dot{m}}_{p}\left(h_{liq}\left(T_{sh}\right)-h_{sol}(T_{ref})\right)$$

**Figure 6:**
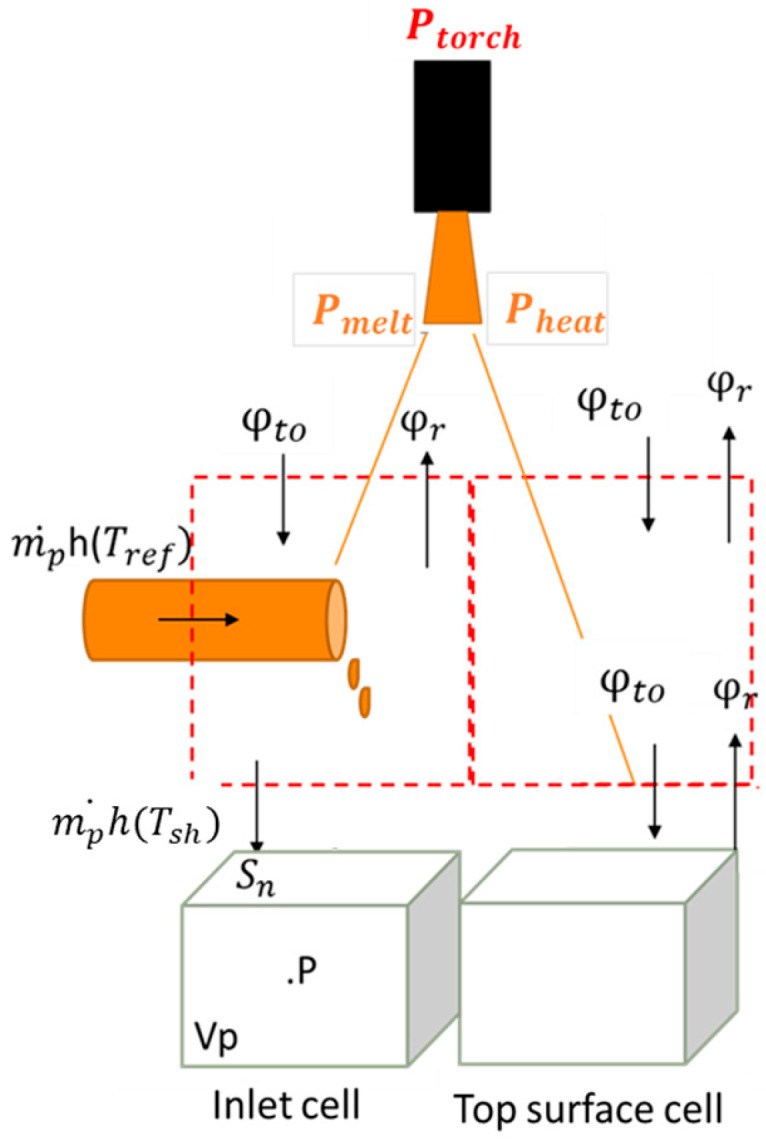
Modeling of the melting rate in each cell of the inlet zone or on the top surface.

The heat flux supplied by the torch minus that lost through radiation is used to heat and melt the solid mass flux, as illustrated in Figure 6. In Equation (8), h_liq_ is defined as the enthalpy of the liquid Ti64 at T_sh_ superheat temperature (inlet value of the model) and h_sol_ is the solid enthalpy at room temperature (25 °C), set to zero as a reference enthalpy in Ansys-Fluent [24]. S_n_ is the north surface of the cell, see Figure 6. 

For the cells where the net heat flux is negative ($\left(\varphi_{to}(x_{p},t)-\varphi_{ray}^{up}(x_{p},t)\right)<0$), no energy is available for melting and ${\dot{m}}_{p}=0$. Otherwise, ${\dot{m}}_{p}$ is calculated from Equation (8).

According this melting model, two source terms are introduced for the cells of the inlet zone, a volumetric mass source term:

(9) $$S_{m,P}=\frac{{\dot{m}}_{p}}{V_{p}}$$

and a volumetric momentum source term:

(10) $$S_{mo,P}=-\frac{{\dot{m}}_{p}^{2}}{\rho_{0}S_{n}V_{p}}$$

where V_p_ is the volume of cell P. The negative sign in Equation (10) comes from the upward pointing *y*-axis.

#### 2.2.4. Trajectory of High-Density Inclusions (HDIs)

Discrete phase trajectories are simulated using a one-way Lagrangian approach. Particle positions are tracked by integrating their velocity ${\vec{u}}_{p}$ over time, which in turn is obtained by integrating their acceleration at every time step. Acceleration is calculated by solving the fundamental equation of dynamics Equation (11) for each particle. In the specific case of HDIs, the lift force can be considered as negligible, so that the equation reduces to:

(11) $$M_{p}(1+\frac{\rho }{2\rho_{p}})\frac{d{\vec{u}}_{p}}{dt}=M_{p}\left(1-\frac{\rho }{\rho_{p}}\right)\vec{g}-C_{D,t}\rho \frac{\pi d_{p}^{2}}{8}\left\|{\vec{u}}_{sl}\right\|\left({\vec{u}}_{p}-\vec{u}\right)+\frac{3}{2}M_{p}\frac{\rho }{\rho_{p}}\frac{d\vec{u}}{dt}$$

where M_p_, d_p_, and ρ_p_ are, respectively, the mass, equivalent diameter, and density of the inclusion. u_sl_ is the sliding velocity between particle and liquid at the particle location. C_D,t_ is a dimensionless drag coefficient. For a particle larger than the Kolmogorov scale η_K_, turbulence modifies the flow around the particle, resulting in a modification of the drag coefficient, which according to Brucato et al. [28] is modeled as:

(12) $$C_{D,t}=C_{D,0}\left(1+8.67\cdot 10^{-4}{\left(\frac{d_{P}}{\eta_{K}}\right)}^{3}\right)$$

C_D,0_ is the drag coefficient of the same moving particle in a stagnant fluid. The latter depends on the particle’s Reynolds number according to the correlation [29]:

(13) CD,0=aRep+bRep2+c

where the values of a, b, and c depend on Re_p_.

Simulation results for the operating conditions of the pilot furnace show that the Kolmogorov scale η_K_ remains between 30 and 400 μm in the upper part of the bath where turbulence is developed. The d_p_ > η_K_ condition is therefore verified. Furthermore, the correction (Equation (12)) becomes significant when $8.67\cdot 10^{-4}{\left(\frac{d_{p}}{\eta_{K}}\right)}^{3}>0.1$, i.e., d_p_ ≳ 5 η_K_.

**Figure 7:**
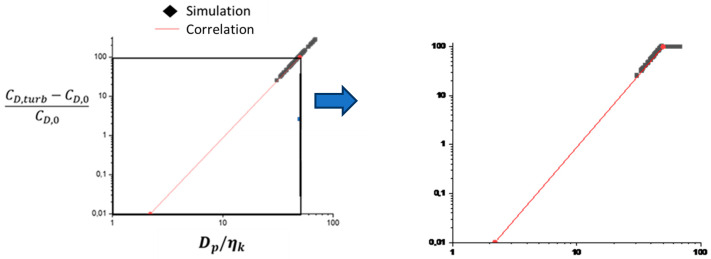
Drag coefficient correction as a function of particle diameter and Kolmogorov scale seen by the particle, given by Brucato [28] (**left**) and applied in PAM3D (**right**).

On the other hand, the correction (Equation (12)) was established and validated by Brucato et al. for an interval of values represented by the black frame on the left in Figure 7. The simulation results reported in Section 3 present conditions that fall outside this validity interval, which led us to limit the correction to the maximum value of $\frac{\left(C_{D,t}-C_{D,0}\right)}{C_{D,0}}=100$, as illustrated in the right side of Figure 7.

#### 2.2.5. Numerical Procedure

**Figure 8:**
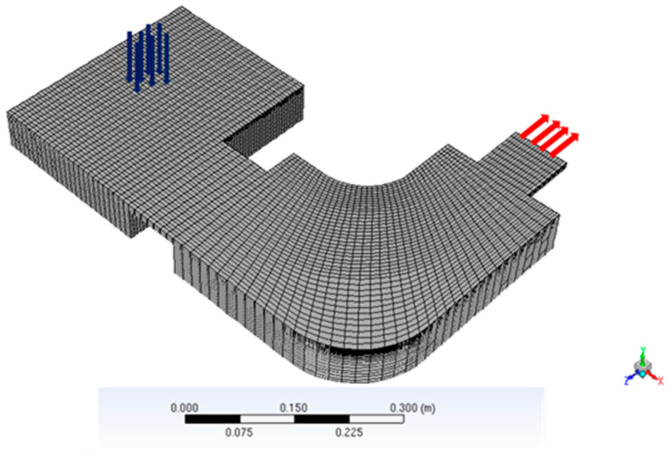
3D mesh of the IRT’Pilot furnace.

The 3D domain is meshed with 74,074 hexahedral cells (see Figure 8). The mesh is refined near to the top surface, with a cell height of 0.87 mm. The solution to the set of transport equations is based on the Ansys-Fluent CFD code V21.1 [24], where several user-defined functions (UDF) have been added to account for all the features of the model. Otherwise, the SIMPLEC algorithm is used to solve the Navier–Stokes equations. In addition, the second-order upwind scheme was used for all the *pde* except for momentum, which uses the first-order upwind scheme. Simulations were run on 8 parallel processing cores, leading to the ratio 1 h computation time per 10 s real operating time. The transient numerical simulation with a time-step of 5 ms is carried on until a quasi-stationary state is reached over a torch period (15 s in the actual case). By this time, the thermal and flow conditions in the crucibles have lost the memory of the initial conditions.

## 3. Results and Discussion

### 3.1. Heat Transfer and Fluid Flow

**Figure 9:**
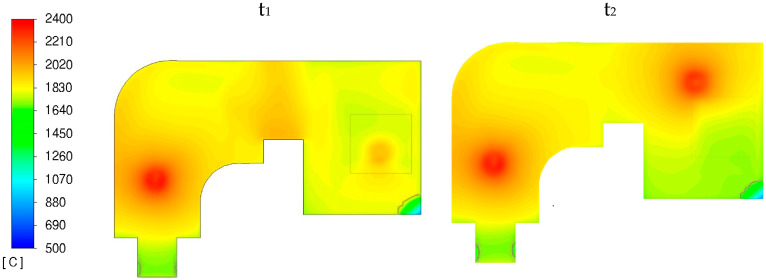
Surface temperature at two different times t_1_ (**left**) and t_2_ (**right**).

Figure 9 shows temperature maps of the PAMCHR crucible surface at two times (t_1_ and t_2_), corresponding to different torch locations. At t_1_, when the melting torch is above the feed surface, shown as a black outline in Figure 9; overheating is not very pronounced since a significant proportion of the torch power is consumed in melting the loaded titanium and not in heating the bath surface. However, when the melting torch leaves the feed surface at time t_2_, a localized zone of higher superheat appears around the torch impact point. Outside the torch impact area, the bath surface temperature averages 1850 °C. We can also see that on the surface of the crucibles, the metal is liquid except on the outlet channel and at the edge of the melting crucible, as shown by the T_liq_ isovalue plotted in black.

**Figure 10:**
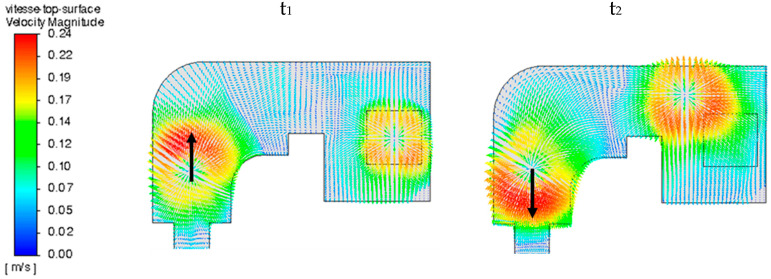
Surface fluid flow at two different times t_1_ (**left**) and t_2_ (**right**).

The plasma blast, combined with the thermal Marangoni effect, generates a strong surface flow, with liquid velocities reaching over 20 cm/s, as shown in Figure 10. This kinetic energy is rapidly degraded by turbulence and viscous stress, so that velocities within the liquid pool remain in the 1 to 5 cm/s range. The bath is therefore moderately turbulent in its upper layer (the first 2 cm near the surface), with a dissipation rate ε_max_ = 1.5 m^2^/s^3^, and a minimum Kolmogorov length scale of 30 μm.

It must be noticed that at t_1_, the torch on the refining hearth goes downstream, whereas at t_2_, this torch moves back upstream, as depicted with the black arrow in Figure 10. Thus, even if the torch blast is modeled without a preferred direction (orthoradial symmetry), a more intense flow upstream from the torch motion is noticed than downstream. This phenomenon can be playfully compared with that of a sheepdog moving through its flock.

### 3.2. Global Balance

When dealing with computational heat and fluid dynamics, an essential factor to consider is the heat balance reported in Table 2. During a torch period, 39% of the electric power is transferred to heat power (280 kW) on the bath surface: 22% of this power is lost to radiation and 58% lost to the side and bottom walls. Lastly, 18% of the heat power is convected by the liquid metal leaving the refining crucible.

### 3.3. Melting Rate

**Figure 11:**
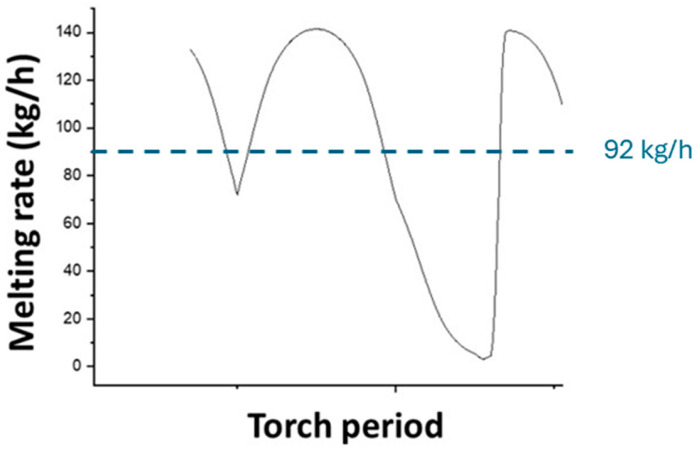
Calculated melting rate over time.

The melting rate is variable over time, depending on the position of the melting torch relative to the material inlet surface. Figure 11 shows the evolution of the melting rate as a function of time over one melting torch period. It varies from 0 when the torch is outside the feed surface to 140 kg/h when the torch is above this surface, with an average value of 92 kg/h, in excellent agreement with the experimental value (92 kg/h).

### 3.4. Liquid Pool Profiles

**Figure 12:**
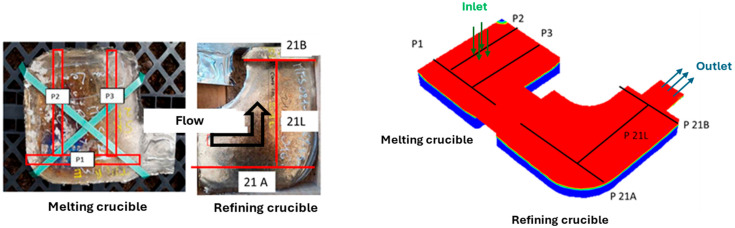
Location of the skull’s sections (**left**) and as they appear in the simulation results (**right**).

The skull was sectioned to compare bath depths. Three sections were made in each crucible, as shown in Figure 12. Planes P1, P2, and P3 are located in the melting crucible, while planes P21A, P21B, and P21L are in the refining crucible.

**Figure 13:**
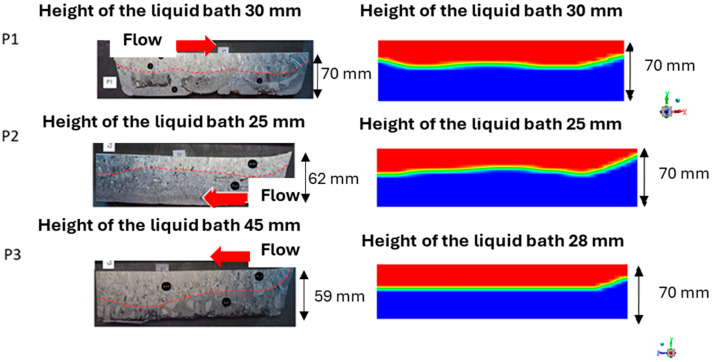
Sections of the melting skull (**left**) and simulated liquid pool (**right**). Flow is given with the red arrow. (Black circular markers locate post-mortem structural or chemical analyses of the alloy). On the right hand side, liquid is in red and solid in blue.

In Figure 13, the solid fronts in planes P1 and P2 of the melting crucible, obtained both experimentally and through PAM3D simulation, exhibit identical shapes, with similar liquid metal depths of 30 mm and 25 mm, respectively. However, plane P3, located near the transfer channel, shows a greater bath depth in the experiment than in the simulation. This discrepancy can be attributed to the torch trajectory—specifically, its inclination—which is not accounted for in the simulation. This results in a shift in the torch’s impact point and an asymmetry in its effect on the bath. Additionally, plane P3 reveals visible differences in the contact quality between the skull and the bottom of the crucible, varying between the left and right sides of the image, which could also contribute to significant variations in bath depth.

**Figure 14:**
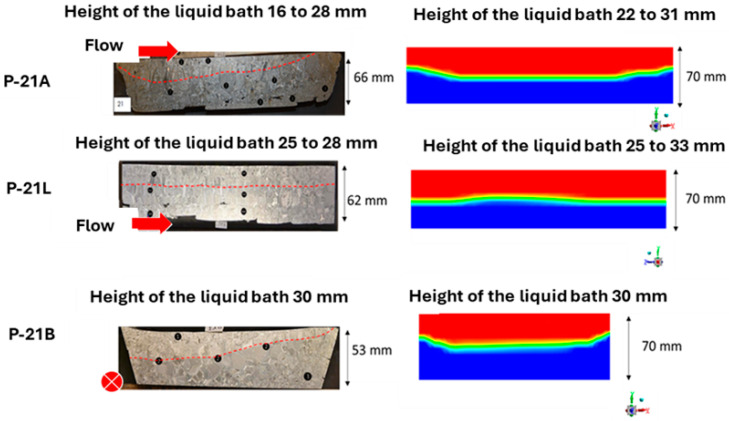
Sections of the refining skull (**left**) and simulated liquid pool (**right**). Flow is given with the red arrow. (Black circular markers locate post-mortem structural or chemical analyses of the alloy). On the right hand side, liquid is in red and solid in blue.

In the refining crucible shown in Figure 14, the measured depths in plane P21L align well with the liquid fractions observed in the PAM3D simulation, showing approximately one-third liquid and two-thirds solid. It is much higher than that obtained in a typical electron beam refining hearth, where this ratio is close to 20% [6], and should have an influence on the residence time of inclusions in the liquid pool. The skull shapes in planes P21B and P21A are less accurately captured. In plane P21A, the simulated bath depth is greater than the measured depth. For plane P21B, while the maximum depths in the simulation and experiment are similar, the experimental pool shape is highly asymmetrical—an aspect not captured in the simulation.

### 3.5. Residence Time Distribution (RTD)

The simulation of copper transport within the process is achieved by coupling the PAM3D code with a simulation using the PAMELA code, which models the growth and solidification of the final ingot. Further details on the PAMELA software can be found in [30]. The copper mass fraction vs. time signal at the refining crucible outlet, calculated by PAM3D, serves as an input condition for the PAMELA model.

**Figure 15:**
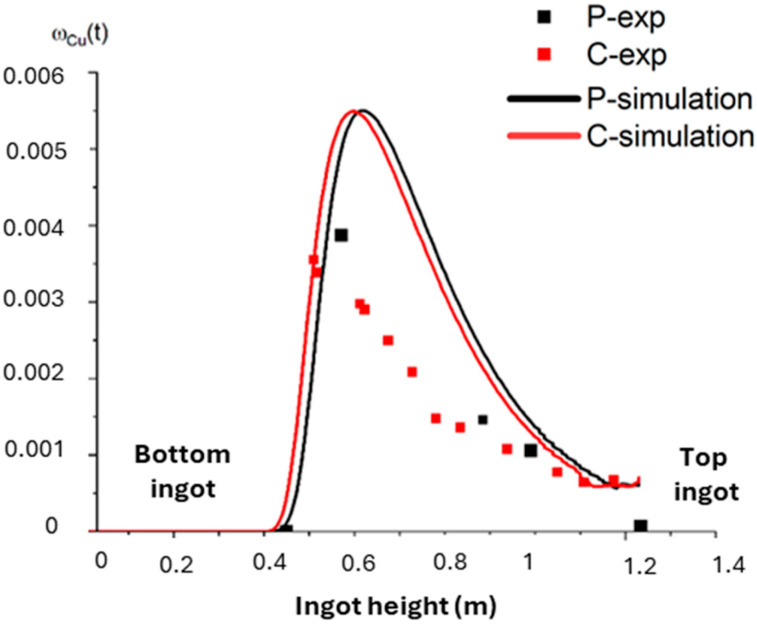
Comparaison of simulated (solid lines) and experimental RTDs (markers). Center of the ingot in red and periphery in black.

The RTD is reported in Figure 15. The solute first appears at the center of the ingot—slightly lower in height than at the periphery—due to the hollowing effect of the liquid pool. While the simulation overestimates the peak mass fraction value and slightly shifts its position within the ingot, the overall shape of the signal remains consistent, aligning with the behavior of perfectly stirred reactors, as described in [23]. The measurements taken at the top of the ingot (i.e., at the end of the trial) show stronger agreement between the experimental and simulated copper fractions. Despite some discrepancies, the experimental and simulated distributions exhibit similar qualitative trends and are sufficiently close quantitatively, particularly at the end of the test. This confirms that the transport mechanisms and thermo-hydrodynamic phenomena are accurately captured by the numerical model.

### 3.6. Fate of HDIs

**Figure 16:**
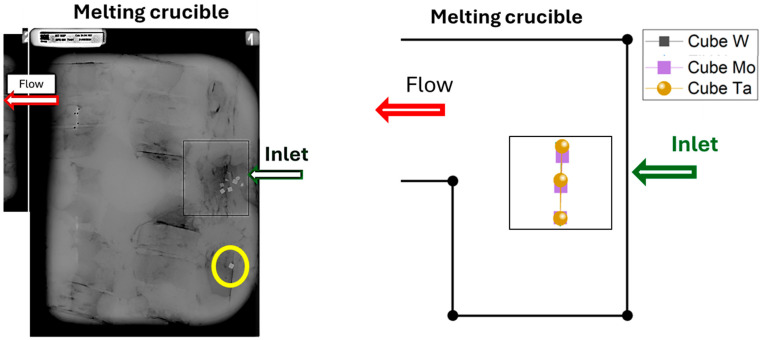
X-ray image of the melting skull (**left**) and calculated location of the trapped HDIs (**right**).

X-ray inspections of the skull were performed using a TC320 RX24 (Cie SGS, Arceuil, France) at a voltage of 270 kV and a current of 12 mA. The resulting X-ray images are shown in Figure 16. The density difference between Ti64 and the HDIs (Mo, W, and Ta) provides sufficient contrast to make the HDIs clearly visible in the images.

All HDIs detected via X-ray are located within the melting crucible, with their trapping positions directly beneath the feeding point—except for one cube, highlighted in yellow in Figure 16, which is slightly displaced from the cluster of other HDIs marked in red. This deviation may be attributed to a slight tilting of the feed box just before melting stopped, causing the cube to be injected slightly farther away.

As expected, due to the significant density difference between the particles and liquid Ti64, the simulated HDIs become trapped by the solid front directly below their injection point. Their residence time in the liquid phase before entrapment is extremely short—less than one second for these 5 mm particles. Calculations varying the HDI size indicate that only particles with an equivalent diameter smaller than 120 μm are transported within the liquid bath. These results agree well with simulations by Xu et al. [11], which also reported residence times of less than one second for particles larger than 1 mm.

## 4. Conclusions and Perspectives

The PAM3D code, developed on the Ansys-Fluent platform, is a phenomenological model that describes the thermal–hydraulic behavior of liquid Ti64 in the melting and refining crucibles of the PAMCHR process. The model for the raw material melting stage employs an enthalpy balance to compute the melting rate, considering the plasma torch’s power and trajectory within the crucible. As in the reality of the industrial process, the melting speed value changes considerably depending on the position of the melting torch, above the raw material inlet zone or outside it. A global heat balance is calculated over time, showing that over 50% of the thermal energy supplied by the torches is lost in the crucible cooling circuit. The behavior of inclusions is simulated using a Lagrangian approach, specifically applied here to HDI refractory inclusions.

A series of tests were conducted using the IRT-M2P’s high-capacity pilot furnace (1.2 MW total electrical power, with a titanium melting rate of up to 150 kg/h) to compare experimental results with numerical simulations. The model accurately predicts the liquid bath profiles, showing a bath depth of approximately one-third of the metal height in the crucible. The residence time distribution of a copper tracer introduced into the raw materials follows a similar trend to the simulation results. Moreover, both the HDI insemination test and the simulation confirm that the process is efficient to remove these particles through sedimentation at the bottom of the melting crucible. This is probably the most important result of this work compared with other processes such as VAR, where efficiency is not totally guaranteed [20,21].

Simulating LDI behavior in the PAMCHR process presents greater complexity, as it requires accounting for dissolution in the Ti64 bath [31,32]. Further experimental and simulation work is planned.

## Figures and Tables

**Table 1:** Coefficients of the relative contributions of the heat provided by the plasma torch [9].

0.66	$\eta_{\mathrm{to}}$
0.05	$\alpha_{\mathrm{élec}}$
0.30	$\alpha_{\mathrm{conv}}$
0.65	$\alpha_{\mathrm{ray}}$

**Table 2:** Global heat balance over a torch period (15 s).

Electrical Power of the Two Torches (kW)	716.59	
Thermal power provided by the two torches on the crucibles (kW)	281.82	
Accumulation (kW)	0.044	~0.01%
Power lost on crucible walls (kW)	163.73	58%
Power lost by heat radiation (kW)	63.08	22%
Enthalpy heat flux transferred to the ingot * (kW)	52.4	18%
Residual	2.57	0.9%

* Enthalpy reference: Solid Ti64 at room temperature.

## Data Availability

The original contributions presented in this study are included in the article. Further inquiries can be directed to the corresponding author.
